# Effect of Metabolic Syndrome on the Functional Outcome of Corticosteroid Injection for Lateral Epicondylitis: Retrospective Matched Case-Control Study

**DOI:** 10.1038/s41598-017-11179-z

**Published:** 2017-09-07

**Authors:** Young Hak Roh, Minjoon Oh, Jung Ho Noh, Hyun Sik Gong, Goo Hyun Baek

**Affiliations:** 1 Department of Orthopaedic Surgery, Ewha Womans University Medical Center, Ewha Womans University College of Medicine, 1071 Anyangcheon-ro, Yangcheon-gu, Seoul 07985 South Korea; 2 Department of Orthopaedic Surgery, Kangwon National University Hospital, 156 Baengnyeong-ro, Chuncheon-si, Gangwon-do 200-722 South Korea; 3 Department of Orthopaedic Surgery, Seoul National University College of Medicine, Seoul National University Bundang Hospital, 173 Gumi-ro, Bundang-gu, Sungnam 13620 South Korea; 4 Department of Orthopaedic Surgery, Seoul National University College of Medicine, Seoul National University Hospital, 101 Daehak-ro, Jongno-gu, Seoul 03080 South Korea

## Abstract

Both obesity and diabetes mellitus are well-known risk factors for tendinopathies. We retrospectively compared the efficacy of single corticosteroid injections in treating lateral epicondylitis in patients with and without metabolic syndrome (MetS). Fifty-one patients with lateral epicondylitis and MetS were age- and sex-matched with 51 controls without MetS. Pain severity, Disability of the Arm, Shoulder, and Hand score, and grip strength were assessed at base line and at 6, 12 and 24 weeks post-injection. The pain scores in the MetS group were greater than those in the control group at 6 and 12 weeks. The disability scores and grip strength in the MetS group were significantly worse than those of the control group at 6 weeks. However, there were no significant differences at 24 weeks between the groups in terms of pain, disability scores and grip strengths. After 24 weeks, three patients (6%) in the control group and five patients (10%) in the MetS group had surgical decompression (p = 0.46). Patients with MetS are at risk for poor functional outcome after corticosteroid injection for lateral epicondylitis in the short term, but in the long term there was no difference in outcomes of steroid injection in patients with and without MetS.

## Introduction

Lateral epicondylitis (LE) is the most common cause of elbow and forearm pain in adults, and it has an annual incidence of 1% to 3% in the general population^1, 2^. Although some patients with symptoms of a shorter duration may resolve spontaneously^3^, the average duration of symptoms for patients with LE is reportedly 44 months^1^ and no difference has been observed in the severity of symptoms in patients that had pain greater or less than 12 months^4^. Despite the high incidence of LE, optimal management for LE has not been established, and numerous treatment options, including bracing, therapeutic exercise, shock wave or ultrasound therapy, corticosteroid injection, and surgical decompression have been used to treat this condition^5–9^. Of these treatment choices, local corticosteroid injections are extensively administered as easy, cost-effective short- to mid-term treatment for patients with LE^10–12^. However, there is limited evidence on the long-term success of corticosteroid injection to treat LE^13–15^, and the case for corticosteroid being superior to wait and see at longer-term follow-up is not solid^13, 14^. The long-term improvement after a corticosteroid injection for LE might in fact be explained by the natural course of the condition^2, 3^. However, some patients need surgical treatment because conservative treatments fail to adequately alleviate the symptoms. The differences in treatment outcomes may, in part, be due to differences in patient characteristics, such as combined medical conditions affecting the individual.

Metabolic syndrome (MetS) is a constellation of medical conditions arising from insulin resistance and abnormal adipose deposition and function^16^. The prevalence of MetS has increased due to the aging population and the obesity epidemic in industrialized countries, resulting in this condition posing a major public health challenge^17, 18^. Both obesity and diabetes are well-known risk factors for tendinopathies^19^, and the prevailing hypotheses of tendon damage in obese subjects indicate two mechanisms: (i) an increased force on load-bearing tendons and (ii) biochemical alterations attributed to systemic bioactive factors^19, 20^. Obesity and diabetes share common pathologic pathways characterized by an increase in cross-linking between collagen fibrils mediated by advanced glycation end products (AGEs) and low-grade inflammation, which amplify the deleterious effect of tendon overuse^21^. The deposit of fat or cholesterol byproducts in tendon tissues can weaken the mechanical strength of the tendon and can further impair healing^22, 23^. Thus, the co-occurrence of MetS with these diseases might affect treatment outcomes of local corticosteroid injections in LE.

Due to the increased prevalence of MetS and the higher incidence of LE, it is important to assess the outcomes of a corticosteroid injection for LE in patients with MetS. Therefore, the aim of this study was to compare the outcome of a corticosteroid injection in patients with or without MetS. We hypothesized that patients with LE and MetS would show decreased functional scores for both objective and subjective measures of function compared to patients without MetS for 24 weeks after corticosteroid injection.

## Materials and Methods

Patients diagnosed with LE and treated with corticosteroid injection between June 2013 and May 2015 were retrospectively enrolled in this study. The inclusion criteria were LE symptoms for more than 3 months, with LE defined as pain on the lateral side of the elbow, pain at the lateral epicondyle region on resisted extension of the wrist, and tenderness at the lateral epicondyle on direct palpation. The exclusion criteria were age younger than 18 years, corticosteroid injection within the past 6 months, previous surgery on the involved elbow, inflammatory diseases (e.g., rheumatoid arthritis or psoriatic arthritis), neck or shoulder pain on the ipsilateral side, and inability to complete a self-reported questionnaire. Among patients without these factors, we identified 51 patients with LE and MetS who were matched for age and sex with 51 controls without MetS. Controls were selected by cumulative sampling method at the end of the follow-up period according to matching variables in a stepwise fashion, first attempting to match on age and then sex. The diagnosis of MetS was made according to the National Cholesterol Education Program (NCEP) Adult Treatment Panel (ATP) III definition^16^, which has been widely used because of its simplicity as its components are easily and routinely measured in most clinical and research settings^24^. MetS was defined by the presence of at least three of the following five criteria: (1) a clinical diagnosis of diabetes treated with oral hypoglycemic medication or insulin or a fasting serum glucose level of 110 mg/dL or higher ; (2) arterial blood pressure of 130/85 mm Hg or higher or current use of antihypertensive medication ; (3) plasma triglyceride level of 150 mg/dL or higher ; (4) high-density lipoprotein cholesterol level of less than 50 mg/dL for females or less than 40 mg/dL for males ; or (5) a waist size greater than 88 cm for females or 102 cm for males. The modified NCEP ATP III criteria suggested that the cut-off points for the waist circumference should be specific according to ethnicity. In individuals of Asian origin, a cut-off of 90 cm in men and 80 cm in women is used^25^. For the NCEP criteria, abdominal obesity is a component of the syndrome, but not a prerequisite for its diagnosis. This study was approved by the institutional review boards of Gil Medical Center, and all participants provided written informed consent. This study did not require any deviation of the current clinical practice and was conducted in accordance with the principles of research involving human subjects as expressed in the Declaration of Helsinki (64th, 2013) and with Good Clinical Practice standard. This study was registered (30th June 2017) at the Clinical Research Information Service (CRiS), Republic of Korea (KCT0002367).

The corticosteroid injection was made with a 23-gauge needle by placing the needle under the extensor origin and injecting with multiple passes of the needle in a fanlike fashion in the area. The content was injected in the deepest aspects of the common tendon origin to limit the risk of skin atrophy. The corticosteroid injection treatment was carried out with a single 2 mL injection, containing 1 mL of lidocaine (10 mg/mL) and 1 mL of triamcinolone acetonide (40 mg/mL). After the injection, the patient was instructed to resume activity as tolerated. No further physical therapy or splinting was recommended, and no patients were injected a second time. The decision to undergo surgery was a mutual decision between the patient and the surgeon.

Patients returned for their functional assessments at 6 (range, 5–8), 12 (11–14) and 24 (22–26) weeks after the corticosteroid injection. The maximum pain-free grip strength was measured in kilogram-force using a Jamar dynamometer (Asimow Engineering, Los Angeles, CA) with the shoulder and forearm in neutral rotation. The mean value of 3 efforts was calculated and included in the analysis, and the dynamometer was calibrated according to a standard procedure by using standardized test weights. The severity of pain during daily activity was evaluated using an 11-point numeric rating scale (NRS), with ‘0’ indicating that the patient experiences no pain and ‘10’ indicating the highest imaginable pain. The patient-reported outcome was evaluated using the Disabilities of the Arm, Shoulder and Hand (DASH) score, which is a self-administered, upper-extremity specific questionnaire that consists of 30 questions^26^. It includes physical functions, symptoms, and social-role function, work, sleep, and confidence items. Five responses are provided per question and are scored from ‘1’ (without difficulty or no symptom) to ‘5’ (unable to engage in activity or very severe symptoms). Thus, the DASH score provides a best possible score of ‘0’ and a worst possible score of ‘100’. The DASH evaluation is user-friendly, reliable, and valid for a range of upper-extremity disorders^27, 28^. The investigators (trained nurse) checked all returned questionnaires for completion, and the participants were assisted in completing the missing items.

### Statistical analysis

A post hoc power analysis was performed on the DASH scores to determine the minimum number of subjects needed to detect a 10-point difference between the two groups, which would be considered clinically relevant (minimal clinically important difference)^29^. Based on this study’s data, the inclusion of 81 patients with an allocation ratio of 40/41 would provide 84% power and a 2-sided level of 0.05 to detect the clinical relevant differences between the two groups.

Descriptive statistics were used to represent the demographics and clinical characteristics of the patients. The Kolmogorov-Smirnov test was used to identify the normality of the variable distributions, and a *t*-test was used to determine any significant differences between the two groups in terms of continuous variables, and the Chi-squared or Fisher exact tests were used to determine any significant differences in the categorical variables. The outcomes at the follow-up visits were compared with use of two-way ANOVA with repeated measures for time with adjustment of p values by the Greenhouse-Geisser epsilon. Statistical significance was accepted when *p* < 0.05.

### Ethical approval

All procedures performed in this study involving human participants were in accordance with the ethical standards of the institutional and/or national research committee and with the 1964 Helsinki declaration and its later amendments or comparable ethical standards. This study was registered (30th June 2017) at the Clinical Research Information Service (CRiS), Republic of Korea (KCT0002367).

## Results

**Figure 1:**
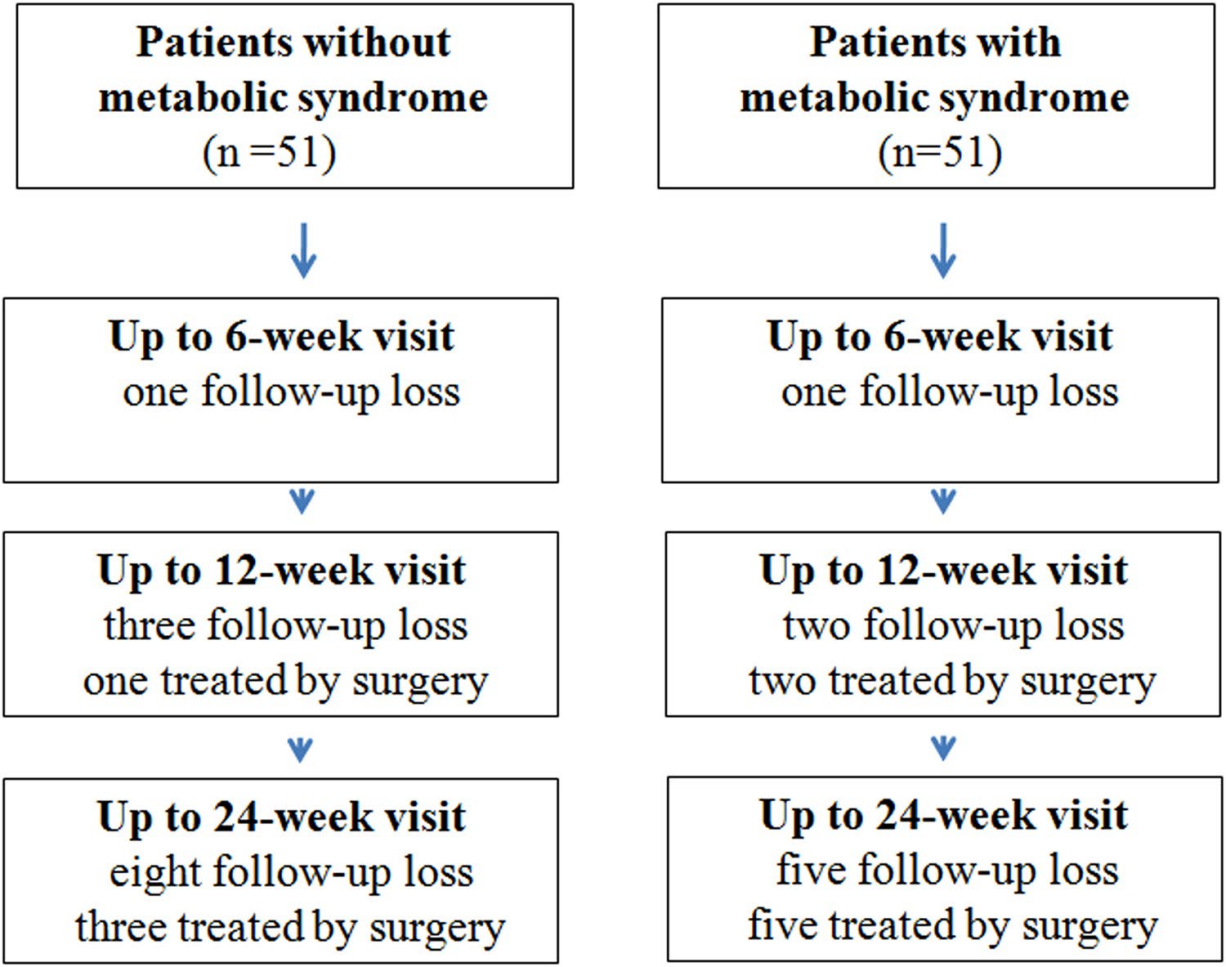
Flow chart of the study population, showing the number of patients with lateral epicondyllitis that were enrolled in the metabolic syndrome group and the control group, and their status at the follow-up visit.

The age and sex of the MetS group were similar to those of the control group, and there was no significant difference in the duration of symptoms and affected side between the two groups. However, the BMI of the patients in the MetS group was significantly greater than that for patients without MetS (Table 1). There were five cases of steroid flare (three in the MetS group and two in the control group) and five cases of skin discoloration/subcutaneous fat atrophy (three in the MetS group and two in the control group), but there were no other serious complications after the injection therapy. Before the 24-week evaluation, 5 patients in the MetS group and 8 in the control group were lost to follow-up (Fig. 1). Missing data were not imputed so as to avoid biasing estimates of the missing outcomes^30^.

**Table 1:** Demographic and clinical characteristics of participants in the study

	Patients without metabolic syndrome (*n* = 51)	Patient with metabolic syndrome (*n* = 51)	*p-*value
Age (years)	45.8 (25–60)	46.1 (26–60)	NS
Female/male	33/18	33/18	NS
BMI (kg/m^2^)	25.3 (18.4–32.5)	30.3 (26.9–36.0)	<0.01
*MetS components*			
Diabetes	5	17	<0.01
Hypertension	11	30	<0.01
Elevated triglyceride	16	47	<0.01
Low HDL	6	31	<0.01
Abdominal obesity	14	46	<0.01
*No*. *of components*			
0	8		
1	24		
2	19		
3		37	
4		10	
5		4	
Symptom duration (months)	10 (4–24)	11 (5–24)	NS
*Affected side*			
Unilateral			
Dominant	28	26	NS
Non-dominant	18	14	NS
Bilateral^*^	5	11	NS
*Baseline measurements* Mean ± SD			
Pain VAS score	5.2 ± 2.1	5.4 ± 2.4	NS
DASH score	39 ± 17	41 ± 18	NS
Grip Strength	21.5 ± 5.9	21.8 ± 6.1	NS

Values are the number (range) unless otherwise indicated. ^*^In case of bilateral involvement, the more severely affected side was chosen for comparative analysis. BMI, body mass index; MetS, metabolic syndrome ; HDL, high density lipoprotein ; SD, standard deviation ; NS, not significant.

**Figure 2:**
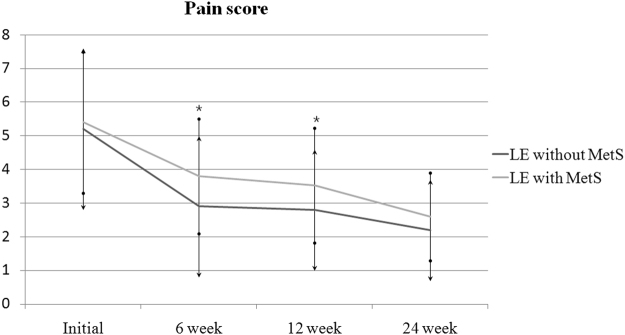
Comparison of pain NRS scores. LE = lateral epicondylitis, MetS = metabolic syndrome, *P < 0.05 vs. the control group at the same time point, error bars = 1 SD. Two-way ANOVA with repeated measures for time with adjustment of p values by the Greenhouse-Geisser epsilon showed significant group effect [*F*(1,79) = 9.37, p = 0.003] and a significant group by time interaction [*F*(2.19, 203.27) = 3.64, p = 0.028].

**Figure 3:**
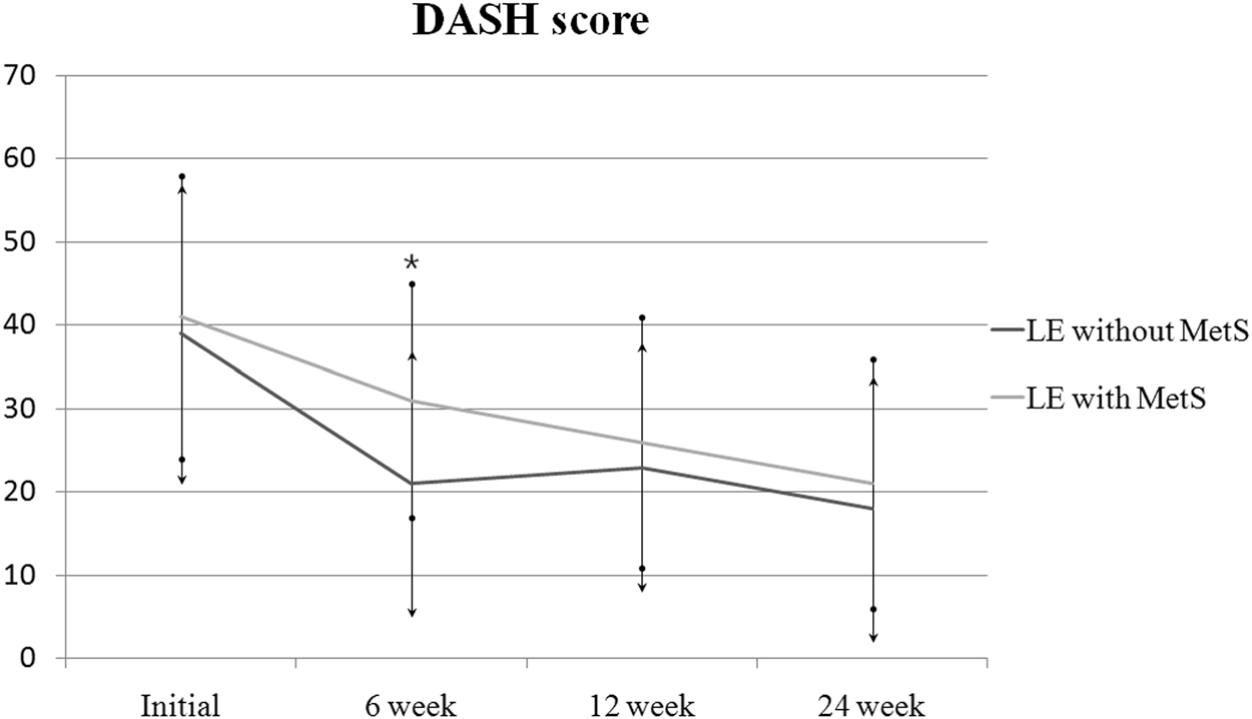
Comparison of DASH scores. LE = lateral epicondylitis, MetS = metabolic syndrome, *P < 0.05 vs. the control group at the same time point, error bars = 1 SD. Two-way ANOVA with repeated measures for time with adjustment of p values by the Greenhouse-Geisser epsilon showed significant group effect [*F*(1,79) = 10.73, p = 0.002] and a significant group by time interaction [*F*(2.48, 195.21) = 3.71, p = 0.026].

**Figure 4:**
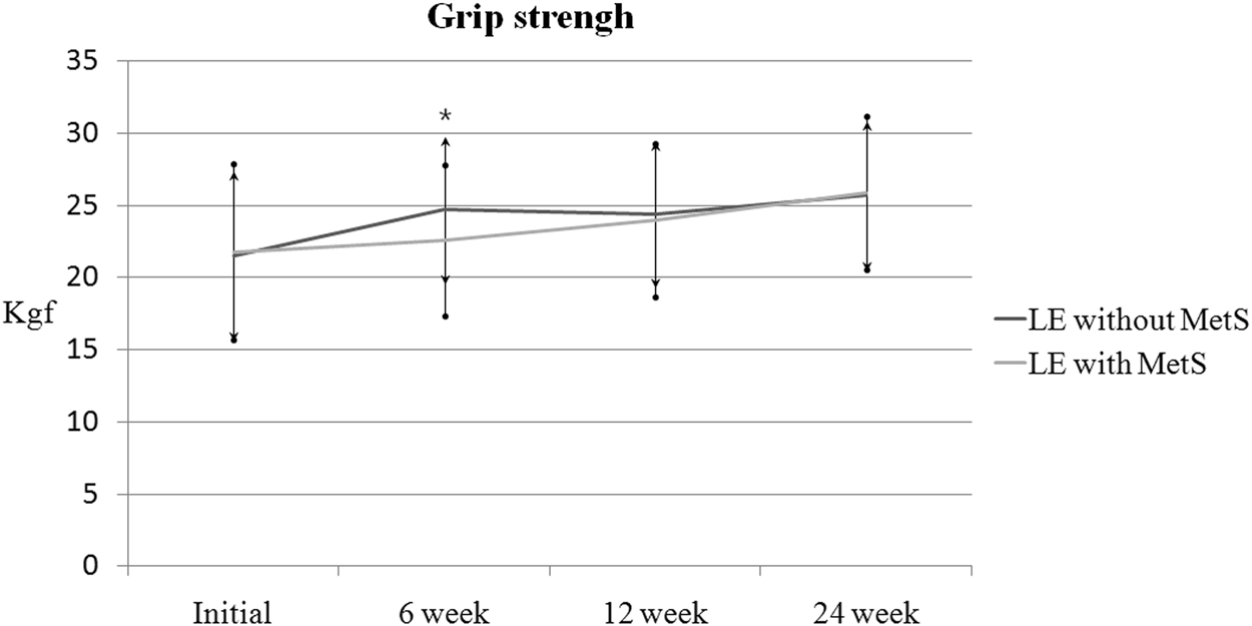
Comparison of grip strength. LE = lateral epicondylitis, MetS = metabolic syndrome, *P < 0.05 vs. the control group at the same time point, error bars = 1 SD. Two-way ANOVA with repeated measures for time with adjustment of p values by the Greenhouse-Geisser epsilon showed no group effect [*F*(1,79) = 2.82, p = 0.097], but a significant group by time interaction [*F*(2.29, 179.21) = 3.25, p = 0.041].

Patients with MetS had similar pre-injection mean grip strengths, pain NRS and DASH scores compared to the control group. Repeated-measures analysis of variance was used to test for a treatment effect, that is, for any average difference between groups in the pattern of change over time. Analysis of pain NRS score showed significant group effect [*F*(1,79) = 9.37, p = 0.003] and a significant group by time interaction [*F*(2.19, 203.27) = 3.64, p = 0.028]. The pain NRS scores in the MetS group were greater than those in the control group at 6 and 12 weeks of follow-up (*p* = 0.02 and 0.04, respectively, Fig. 2). Similarly, analysis of pain DASH scores showed significant group effect [*F*(1,79) = 10.73, p = 0.002] and a significant group by time interaction [*F*(2.48, 195.21) = 3.71, p = 0.026]. The DASH scores in the MetS group were significantly greater (with more severe symptoms) than those of the control group at 6 weeks of follow-up (*p* = 0.01, Fig. 3). Analysis of grip strength showed no group effect [*F*(1,79) = 2.82, p = 0.097], but a significant group by time interaction [*F*(2.29, 179.21) = 3.25, p = 0.041]. The grip strength was weaker in patients with MetS than in the control group at 6 weeks of follow-up (*p* = 0.04, Fig. 4). However, there were no significant differences at the final follow-up between the MetS and control group in terms of the pain NRS scores, DASH scores and grip strengths. After 24 weeks of follow-up, three patients (6%) in the control group and five patients (10%) in the MetS group had surgical decompression due to persistent or recurrent symptoms after the injection (*p* = 0.46).

## Discussion

The most important finding of the present study was that corticosteroid injections in MetS patients were not as effective in the short-term with regard to subjective pain perception and functional recovery. However, there were no significant differences in pain, grip strength, and functional score between the two groups at the end of the study (24-week follow-up). At 6 and 12 weeks, the pain improvement was better in the control group, while at 6-week follow-up the DASH and grip strength was better in the control group.

MetS comprises a cluster of risk factors for large-vessel artherosclerotic disease^16^, and diabetes is a well-known risk factor for tendinopathies^31, 32^. Hyperglycaemia associated with diabetes alters tendon homeostasis^33, 34^, affects collagen cross-linking in tendons^35^ and reduces proteoglycan content^36^. Advanced glycation end-products (AGEs) also diminish tendon collagen fiber sliding^19, 37^. Obesity has also been recognized as a risk factor for tendinopathy in non-load-bearing as well as load-bearing tendons^38, 39^. The probability of tendon abnormalities is higher in males with an increased waist circumference, and asymptomatic Achilles tendon pathology is reportedly associated with central fat distribution in males^21, 40^. Hypercholesterolemia results in fatty deposits in muscle and tendon tissues as well as vascular endothelial cells or liver cells^41, 42^, and microscopic cholesterol deposition inside the tendons could initiate and maintain low-grade persistent inflammation, which may be responsible for chronic tendon degeneration^43^.

Lateral epicondylitis is thought to be related to the overuse of the extensor carpi radialis brevis muscle, producing pain in the lateral elbow and forearm region^2^. Although the role of inflammation in the pathophysiology of this condition is questionable, lateral epicondylitis is postulated to involve degenerative changes in the epicondylar enthesis of the extensor carpi radialis brevis and perhaps also the supporting collateral ligamentous complex and joint capsule^44^. Uncertainty regarding the pathologic basis of lateral epicondylitis underlies, in part, the lack of consensus on its optimal management^13–15^. Some authors have supported corticosteroid injection as an effective non-operative treatment for LE^10–12^, while others have considered the injection provides only temporary relief while waiting for spontaneous recovery^13, 14^. Interestingly, earlier randomized trials were more likely to show significant symptom relief after a steroid injection for LE whereas recent trials tend not to show a significant effect resulting from the steroid injection^2, 45^. A long-term improvement after a corticosteroid injection for LE might be considered as the natural course of the condition because spontaneous resolution has been reported at up to 80% of patients within 1 year^2, 3^. The systematic review from Coombes *et al*.^15^ showed that corticosteroid injection was effective in the short-term, yet produced negative outcomes at 6-months and 12-months.

Even when a corticosteroid injection does not improve long-term outcomes, some patients and clinicians may be unwilling to wait several months to achieve pain resolution and functional improvement, particularly when a timely return to physically demanding work or sport is needed. An observation-only approach may lead to short-term disability and pain, with the corresponding economic cost and lost productivity^46, 47^. Thus, we believe that the use of a corticosteroid injection may depend on the severity and duration of the symptoms.

The variation in treatment outcomes in previous studies might be attributed to differences inherent to the studies, such as severity of the disease, occupational state, and concurrent medical condition of the participants. Female gender, high baseline pain and disability, and long symptom duration have been associated with poor treatment outcomes for LE^48, 49^. Previous studies have demonstrated that the presence of diabetes mellitus is associated with a worse response to corticosteroid injections^50–52^, but studies of patients with medical comorbidities other than diabetes mellitus are lacking. In this study, although significant improvements in symptom severity and hand function similar to those of LE patients without MetS occurred at 24 weeks after corticosteroid injection, the MetS group had worse functional outcomes than those without MetS in terms of pain NRS scores, DASH scores, and grip strength in the early post-injection period (up to 12 weeks).

There were several limitations to our study. First, the assessment included short-term results, which stand in contrast with the chronic nature of LE, and this impedes having a better understanding of the natural course of the pain and function of LE. On the other hand, LE is a self-limiting condition, and functional improvements may be partially attributable to the natural history of the condition. Thus, the main purpose of treatment may be to shorten the duration of the symptoms and disability. Moreover, a 24-week follow-up is believed to be sufficient to evaluate the effect^15^. Second, the pharmacokinetics and effective dosage of locally administered corticosteroid have not been properly established, and the regimen in this study may not be optimal in its effectiveness and safety, even though its administration was determined in previous studies. Third, only one type of questionnaire was used to assess the functional outcomes of the patients. The minimal clinically important differences or responsiveness of the questionnaires after corticosteroid injection may differ between functional assessments, and another type of functional assessment might result in different conclusions. Third, 13 (13%) patients were lost to follow-up before the 6-month evaluation, and there were some missing questions and questionnaires in our cohort. Finally, the patients were limited to one ethnic population drawn from an urban area. Therefore, their characteristics and the study results may not be generalizable to other populations.

## Conclusion

The results of this study suggest that patients with LE and MetS are at risk for higher pain scores and lower functional outcomes after the corticosteroid injection for LE in the short term, but in the long term there was no difference in outcomes of steroid injection in the MetS and non-MetS group.
